# Assessment of Acute Acral Lesions in a Case Series of Children and Adolescents During the COVID-19 Pandemic

**DOI:** 10.1001/jamadermatol.2020.2340

**Published:** 2020-06-25

**Authors:** Juncal Roca-Ginés, Ignacio Torres-Navarro, Javier Sánchez-Arráez, Carlos Abril-Pérez, Oihana Sabalza-Baztán, Sergio Pardo-Granell, Vicent Martínez i Cózar, Rafael Botella-Estrada, Montserrat Évole-Buselli

**Affiliations:** 1 Department of Dermatology, Hospital Universitario y Politécnico La Fe, Valencia, Spain; 2 Department of Microbiology, Hospital Universitario y Politécnico La Fe, Valencia, Spain; 3 Department of Pathology, Hospital Universitario y Politécnico La Fe, Valencia, Spain; 4 Department of Medicine, School of Medicine, Universitat de València, Valencia, Spain

## Abstract

**Importance:**

A novel coronavirus named *severe acute respiratory syndrome coronavirus 2* (SARS-CoV-2) has recently been identified as the cause of a pandemic called *coronavirus disease 2019* (COVID-19). In this context, some associated skin diseases have been described. Cutaneous lesions referred to as *acute acro-ischemia* have been reported as a possible sign of COVID-19 in adolescents and children.

**Objective:**

To evaluate the pathogenesis of these newly described acute acral lesions.

**Design, Setting, and Participants:**

This prospective case series was conducted at La Fe University Hospital, a tertiary referral hospital in Valencia, Spain, between April 9 and April 15, 2020. Among 32 referred patients, 20 children and adolescents with new-onset inflammatory lesions did not have a diagnosis.

**Exposures:**

Patients were not exposed to any drug or other intervention.

**Main Outcomes and Measures:**

We performed reverse transcriptase–polymerase chain reaction for SARS-CoV-2 and a range of blood tests for possible origins of the lesions. Skin biopsies were performed in 6 patients.

**Results:**

Of the 20 patients enrolled, 7 were female and 13 were male, with an age range of 1 to 18 years. Clinical findings fit into the following patterns: acral erythema (6 patients), dactylitis (4 patients), purpuric maculopapules (7 patients), and a mixed pattern (3 patients). None of the patients had remarkable hematologic or serologic abnormalities, including negative antibodies to SARS-CoV-2. Biopsies performed in 6 patients showed histologic findings characteristic of perniosis.

**Conclusions and Relevance:**

The clinical, histologic, and laboratory test results were compatible with a diagnosis of perniosis, and no evidence was found to support the implication of SARS-CoV-2 infection.

## Key Points

**Question:**

What is the association between acute acral lesions and coronavirus disease 2019 (COVID-19) in children and adolescents?

**Findings:**

In this case series of 20 patients aged 1 to 18 years with new-onset acral inflammatory lesions, all lacked systemic manifestations of COVID-19. Both reverse transcriptase–polymerase chain reaction and serologic test results were negative for severe acute respiratory syndrome coronavirus 2.

**Meaning:**

An association between acral skin disease and COVID-19 has yet to be proved.

## Teaser

This case series describes 20 children and adolescents who presented with new-onset acral inflammatory lesions during the COVID-19 pandemic.

## Introduction

At the end of 2019, a novel coronavirus called *severe acute respiratory syndrome coronavirus 2 (*SARS-CoV-2) was identified as the cause of an outbreak of pneumonia and severe acute respiratory syndrome in Wuhan, China.^1^ On January 30, 2020, the World Health Organization declared coronavirus disease 2019 (COVID-19) a public health emergency of international concern.^2^ Although SARS-CoV-2 infection can affect individuals of any age, severe illness is uncommon in children.

Recently, possible COVID-19–related skin changes have been described: mostly urticaria, drug-related eruptions, and chickenpox-like vesicles.^3^ In addition, cutaneous lesions referred to as *acute acro-ischemia* have been reported as a possible sign of SARS-CoV-2 infection in adolescents and children.^4^ In this article, we report an outbreak of acral skin lesions observed between April 9 and April 15, 2020.

## Methods

A prospective case series was performed at La Fe University Hospital, Valencia, Spain, to assess the clinical and etiologic features of children and adolescents with acute acro-ischemia. Among 32 patients referred for acral lesions between April 9 and April 15, 2020, we included 20 who presented with new-onset acral inflammatory lesions without an obvious diagnosis of recognizable cause.

Each patient underwent a complete blood cell count; biochemistry tests for liver and kidney function, erythrocyte sedimentation rate, and levels of ferritin, lactate dehydrogenase, and C-reactive protein; coagulation tests, including levels of D-dimer, cryoglobulins, and proteins C and S; urine sediment examination; autoimmunity tests for antinuclear antibodies (enzyme-linked immunosorbent assay [ELISA] and indirect immunofluorescence assay), antineutrophil cytoplasmic antibodies, antiphospholipid antibodies (lupus anticoagulant, anti–β_2_-glycoprotein, anticardiolipin antibody), C3, C4, and interleukin 6; serologic tests for enterovirus, Epstein-Barr virus, human herpesvirus 6, parvovirus B19, mycoplasma, rubella, and measles; tests for immunoglobulin (Ig) G, IgM, and IgA (COVID-19 ELISA Kit, Vircell; sensitivity and specificity for IgG and IgM + IgA joint detection of 70% and 98%); and reverse transcriptase–polymerase chain reaction (RT-PCR) by nasopharyngeal swab for SARS-CoV-2 (Viasure SARS-CoV-2 Real Time PCR Detection Kit, CerTest Biotec; detection limit ≥10 RNA copies per reaction for the *ORF1ab* and *N* genes).

This study was approved by the institutional review board of La Fe University Hospital, and written informed consent for each procedure and for publication was obtained from all patients or their families.

## Results

Twenty patients were included in this study, 13 of whom were male. Main characteristics of the patients are depicted in the Table.

**Table.** Main Characteristics of the Patients^a^

Patient No.	Sex	PAVR	Wears shoes at home	Heat in home	Co-inhabitants with similar symptoms	Disease duration before consultation, d	Location of skin lesions	Type of acral lesions	Histopathologic findings		
									Epidermal changes	Dermal changes	Infiltrate allocation
1	M	Yes	Yes	No	No	7	Feet	AE	Scattered NKA	Endothelial swelling	Papillary dermis
									Spongiosis		PV
									Mild VC		PE
2	F	Yes	No	No	Yes	30	Hands	D	NP	NP	NP
3	F	Yes	Yes	No	No	9	Hands and feet	MP (D, PMP)	NP	NP	NP
4	M	Yes	Yes	No	No	26	Hands and feet	D	NP	NP	NP
5	M	Yes	Yes	No	No	21	Feet	PMP	Abundant NKA	Endothelial swelling	Papillary dermis
									Spongiosis	Lymphocytic vasculitis	PV
									Severe VC	Fibrin deposition	PE
										Moderate edema	
6	F	Yes	Yes	No	No	10	Feet	D	NP	NP	NP
7	M	No	Yes	No	No	10	Feet	D	NP	NP	NP
8	F	Yes	Yes	No	No	10	Feet	PMP	Abundant NKA	Endothelial swelling	Papillary dermis
									Spongiosis	Lymphocytic vasculitis	PV
									Severe VC	Fibrin deposition	PE
										Moderate edema	
9	M	No	Yes	No	Yes	17	Feet	PMP	NP	NP	NP
10	F	No	No	No	Yes	7	Feet	MP	NP	NP	NP
11	M	No	Yes	No	No	10	Feet	PMP	Mild NKA	Endothelial swelling	Papillary dermis
									Spongiosis	Dermal thrombi	PV
									Mild VC		PE
12	M	No	Yes	No	No	30	Feet	PMP	NP	NP	NP
13	M	No	No	Yes	No	10	Hands and feet	AE	NP	NP	NP
14	M	No	Yes	No	No	3	Feet	AE	NP	NP	NP
15	M	No	No	No	Yes	7	Feet	MP	NP	NP	NP
16	M	No	Yes	Yes	No	7	Feet	PMP	Mild NKA	Endothelial swelling	Papillary dermis
									Spongiosis	Lymphocytic vasculitis	PV
									Severe VC	Moderate edema	PE
17	F	Yes	Yes	No	No	4	Hands and feet	AE	NP	NP	NP
18	F	No	No	No	Yes	14	Hands	AE	NP	NP	NP
19	M	Yes	Yes	No	Yes	14	Feet	AE	NP	NP	NP
20	M	No	Yes	No	No	19	Feet	PMP	Abundant NKA	Endothelial swelling	Papillary dermis
									Spongiosis	Fibrin deposition	PV
									Mild VC	Dermal thrombi	PE

Abbreviations: AE, acral erythema; D, dactylitis; MP, mixed pattern; NKA, necrotic keratinocytes; NP, not performed; PAVR, previous acral vascular reactivity; PE, perieccrine; PMP, purpuric maculopapules; PV, perivascular; VC, vacuolar changes.

^a^ Ages ranged from 1 to 18 years.

No patient had any clinical symptoms (fever, fatigue, dry cough, anorexia, myalgia, dyspnea, sputum, headache, sore throat, smell or taste disorders, or rhinorrhea) suspected to be COVID-19–related.^1,2,5^ Similarly, no co-inhabitant showed any symptoms. Ten of 20 patients lived with relatives older than 50 years (6 of whom were older than 80 years). The mean (SD) age of the patients was 12.3 (4.3) years, and no patient was older than 18 years. Nine of them (45%) had a history of vascular reactive disease of the hands (Raynaud phenomenon or perniosis). Only patient 8 had a history of connective tissue disease (systemic lupus erythematosus). None of them reported previous drug intake except for patient 18, who had taken a single dose of 500 mg of acetaminophen 2 weeks before, and patient 10, who had started treatment for anemia with ferric sulfate 1 month before. Fifteen (75%) reported walking barefoot around the house during the quarantine. Of all patients, only 2 lived in a home equipped with a heating system. No abnormal test results were found in any patient except for patient 3 and patient 8, who had positive results for antinuclear antibodies (titers of 1/160 and 1/1280, respectively).

**Figure 1.**
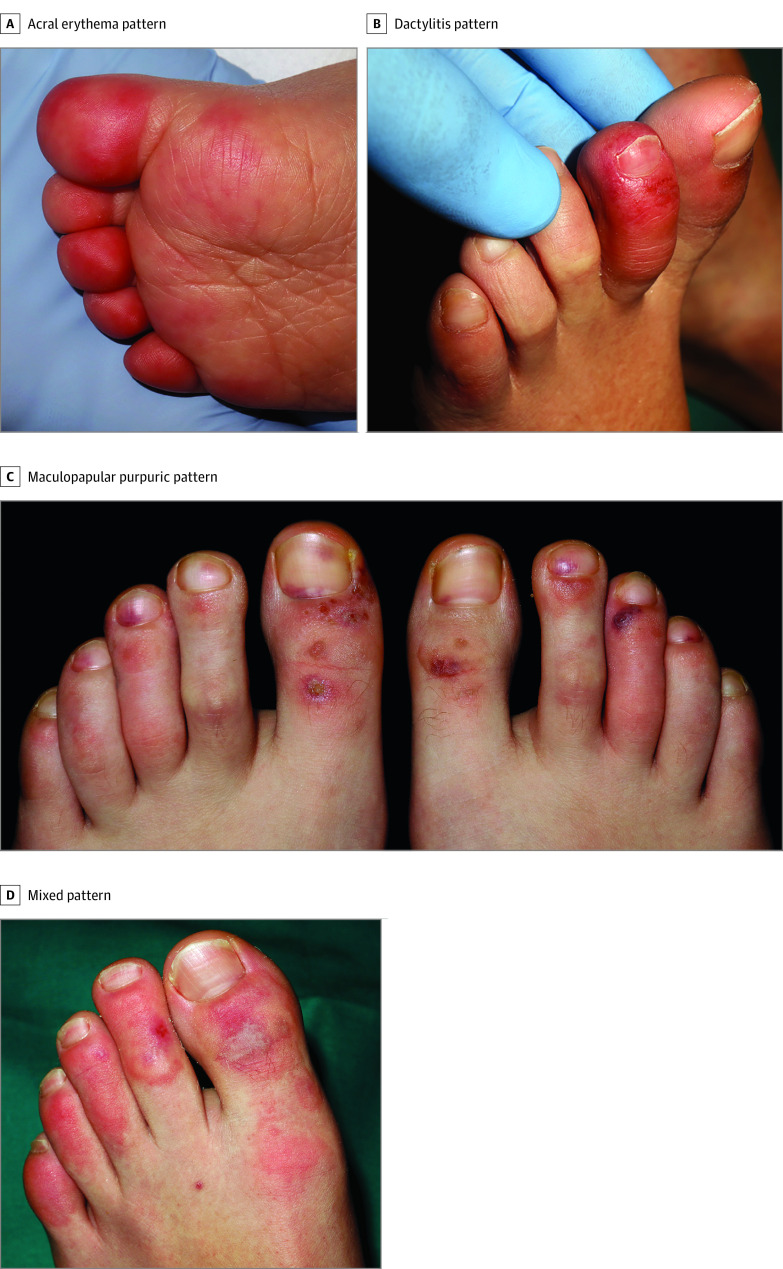
Details of the Clinical Spectrum A, Acral erythema pattern on the dorsal side of the toes. B, Inflammation of 1 toe showing a dactylitis pattern. C, Moderate vasculitic-like lesions on the feet demonstrating a maculopapular purpuric pattern. D, Mixed pattern composed of dactylitis and purpuric maculopapules.

Dermatologic findings were classified into the following groups based on skin pattern: periungual erythema; inflammation of 1 or more fingers with occasional whitish areas, which we have called *dactylitis*; and purpuric maculopapules with occasional blisters. In some patients, we observed a mix of lesions. Acral erythema was found in 6 (30%) of the cases (Figure 1A), dactylitis in 4 (20%) (Figure 1B), purpuric maculopapules in 7 (35%) (Figure 1C), and a mixed pattern in 3 (15%) (Figure 1D).

**Figure 2.**
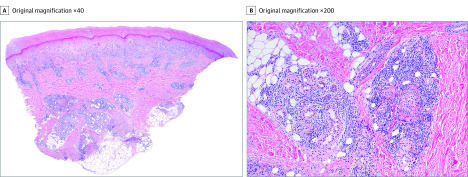
Main Histologic Features A, Acral skin with moderate edema in the papillary dermis, perivascular/perieccrine lymphohistiocytic infiltrate, and lymphocytic vasculitis (hematoxylin-eosin). B, Severe perieccrine and deep perivascular infiltrate. Notice the presence of lymphocytic vasculitis as well as fibrin deposition in the vessel walls (hematoxylin-eosin).

We obtained 6 skin biopsy specimens from 6 different patients. Results are presented in the Table and Figure 2. In all patients, results of serologic and viral testing were negative for SARS-CoV-2 as well as other viruses.

## Discussion

On March 14, 2020, a state of emergency was declared in Spain, and strict stay-at-home rules were imposed. Citizens were alerted to a series of signs and symptoms to detect early SARS-CoV-2 infection. Reports of diverse cutaneous lesions as possible symptoms of COVID-19 have led to an increase in visits to our hospital for any of these manifestations.^4^ However, apart from temporal coexistence, the involvement of COVID-19 in the development of these lesions has not been proved so far.

In this article, we report a series of 20 cases of cutaneous lesions classifiable on the clinical spectrum of perniosis. Regarding histologic features, it is worth noting that there is bias because 5 of 6 biopsies were performed in patients with more severe lesions. Nevertheless, the histologic findings in all of them were characteristic of chilblains, confirming the clinical impression.^6^

When evaluating the pathogenesis of these lesions, several possibilities emerge. Both our cases and others reported in the literature have developed in a short space of time, generally with an onset in the second to third week of the pandemic.^7,8,9,10,11,12^ Some of the cases that have been described occurred in patients with SARS-CoV-2 infection demonstrated by RT-PCR or in symptomatic patients, but there are also a large number of patients, similar to ours, in whom the presence of the virus could not be demonstrated by RT-PCR, the serologic test results were negative, or the patients were asymptomatic.^7,8,9,10,11^

At least 3 different scenarios may be considered to explain the abrupt appearance, during the peak of the pandemic, of these characteristic lesions in a group of SARS-CoV-2–negative patients. One possibility is that the patients were in a very early stage of the disease, which would explain the negativity of PCR and serologic test results.^8^ This seems to us the least probable explanation, given that the mean (SD) duration of the disease before consultation in our series was 13.25 (8.11) days and we conducted follow-up in all patients for 4 additional weeks.

The second alternative is that acrocyanosis and perniosis were a subacute manifestation of the infection, in which patients no longer had detectable viral particles.^8,9,10,11,12^ Additionally, in situations in which the viral inoculum was small, it is conceivable that the RT-PCR results were negative, patients did not develop other symptoms, and the serologic response was of low intensity and not detectable with the tests currently available. Serologic responses have been shown to be lower in young individuals than in older ones.^13^ In this scenario, the only manifestations of COVID-19 could be endotheliitis and a facility for thrombosis in the distal small vessels of the extremities.^8,9,14^ Entotheliitis and thrombosis have been described in patients with severe COVID-19 with previous endothelial damage and cardiovascular comorbidity (eg, diabetes, hypertension, obesity).^14,15^ However, the absence of those risk factors and the unaltered results of the coagulation tests performed in our patients do not support this explanation.

Finally, a third possibility, which is not supported by the results of all of the complementary examinations carried out in our patients, is that these skin lesions are not induced by the virus but by the quarantine state itself. Accordingly, this quarantine perniosis appeared mainly in children isolated in houses that were not well suited for individuals who spent long periods barefoot or only wearing socks and with very little physical activity.

### Limitations

This study was carried out in a short period and with patients from a single center. Furthermore, there is still limited knowledge regarding the clinical manifestations of and detection methods for SARS-CoV-2.

## Conclusions

In this case series of 20 children and adolescents, a relationship between acute acral skin changes and COVID-19 could not be demonstrated. Other studies with improved microbiologic tests or molecular techniques aimed at demonstrating the presence of SARS-CoV-2 in the skin may help to clarify this problem.
